# Problems Using Data Gloves with Strain Gauges to Measure Distal Interphalangeal Joints’ Kinematics

**DOI:** 10.3390/s22103757

**Published:** 2022-05-15

**Authors:** Alba Roda-Sales, Joaquín L. Sancho-Bru, Margarita Vergara

**Affiliations:** Departamento de Ingeniería Mecánica y Construcción, Universitat Jaume I, E12071 Castellón de la Plana, Spain; rodaa@uji.es (A.R.-S.); vergara@uji.es (M.V.)

**Keywords:** data glove, hand, biomechanics, hand kinematics, interphalangeal joints, strain gauge

## Abstract

Data gloves with strain gauges are a widely used technology to record hand kinematics. Several researchers have experienced problems when using data glove models to record distal interphalangeal (DIP) joints, mainly in relation to bad glove fitting. The aim of this work is to report the problems that arise when using one of these gloves (CyberGlove) and to determine an appropriate hand size to avoid these problems. First, static controlled postures of DIP joints and dynamic recordings while closing/opening the fist were taken using the data gloves on participants with different hand sizes, in order to establish the minimum hand length that does not pose recording problems. The minimum obtained hand length that allowed proper recording was 184 mm. Then, validation was performed, which consisted of recording the functional range of motion of the DIP joints in a sample of eight healthy participants with hand lengths longer than the minimum obtained one. These results were then compared to the results found in the literature. Although the glove fit properly, some problems remained: difficulty to record small flexion angles or a diminished touch sensitivity. Its usability would improve if two or three different glove sizes were commercially available.

## 1. Introduction

The human hand consists of many small interconnected bones to provide more than 25 main degrees of freedom (DoF), which makes recording hand kinematics a complex task. Several hand motion capture techniques have been reported in the literature, such as optical systems with reflective markers, which are considered the gold standard and allow recordings with positioning errors < 2 mm [1]. Another popular optical motion capture system is the Leap Motion Controller, a non-marker-based alternative with a simple set up. Nevertheless, its positioning reconstruction is not accurate enough for hand kinematics analysis applications, with discrepancies with marker-based systems of 17.3 ± 9.56 mm [2], although several works in the literature obtained higher positioning precision when using several Leap Motion Controllers simultaneously [3]. However, occlusion problems hinder the use of all optical systems during product manipulation recordings. For this reason, data gloves are a widely used alternative [4] because they are simple to set up and these occlusion problems do not arise. The most widely implemented technology on these gloves is the measurement with strain gauges, but other alternatives such as inertial sensors have been used for this purpose [5]. In data gloves using strain gauges, each gauge has its specific location in the glove to measure the bending angle that corresponds to the rotation in a specific DoF. For best accuracy purposes, gloves need to be calibrated every time they are put on a subject to obtain anatomical angles from gauge data. The calibration procedures combine manual and automatic methods [6]. Manual methods consist in placing the joints at specified angles while measuring from the glove and obtaining gains from regressions. Automatic methods can be: (i) open-loop kinematic methods, which minimize the error in the posture either because subjects perform a known path or through comparison with data from another motion capture system, and (ii) closed-loop kinematic ones, which use optimization methods to obtain the gains that best assure a closed kinematic chain. Nevertheless, using an across-subject calibration protocol may speed up recording sessions and data post-processing with mean errors < 4.5° [7].

**Figure 1:**
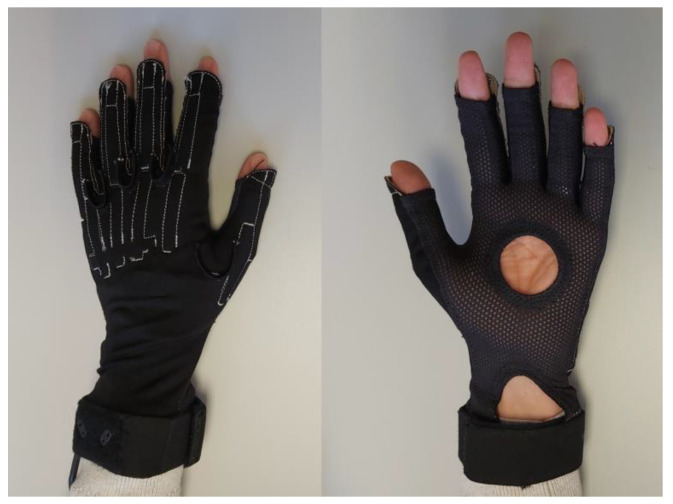
Dorsal (**left**) and palmar (**right**) views of the CyberGlove data glove.

One of the most popular commercially available data gloves is CyberGlove (CyberGlove Systems LLC, San Jose, CA, USA), which has been used in fields such as ergonomics of hand tools [8], hand kinematics in activities of daily living (ADLs) [4,9,10], functional assessments [11], virtual rehabilitation [12], and sign language recognition [13], among others. This glove is made of synthetic elastic mesh fabric on the palmar side, and a denser synthetic elastic fabric on the back, where extensometric gauges and wiring are embedded (Figure 1).

**Figure 2:**
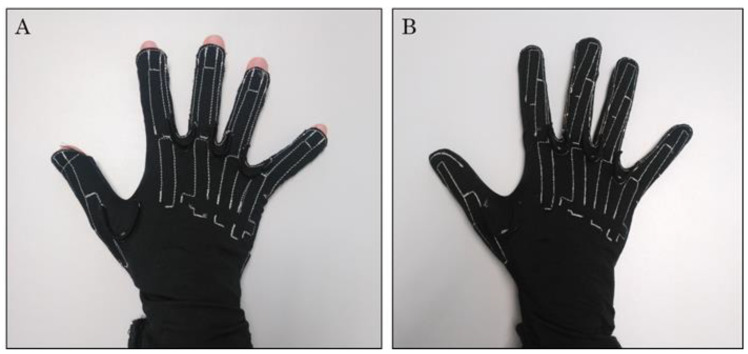
CyberGlove models: (**A**) 18 DoF model and (**B**) 22 DoF model.

One CyberGlove model is equipped with 18 gauges and another with 22. Both gloves record flexion of proximal interphalangeal (PIP) and metacarpophalangeal joints of index to little fingers (8 DoF), abduction between fingers (3 DoF), thumb carpometacarpal abduction and flexion (2 DoF), flexion of thumb metacarpophalangeal and interphalangeal joints (2 DoF), palmar arch (1 DoF), and wrist flexion and radioulnar deviation (2 DoF). The model with 22 gauges has 4 additional gauges to record the flexion/extension of distal interphalangeal (DIP) finger joints (Figure 2).

The fingertips of the 18 DoF model are not covered, allowing tactile feedback during object manipulation. Contrarily, the fingertips of the 22 DoF model are covered so that glove fingers are slightly longer to accommodate big hands, which, depending on hand size, may pose problems with improper glove fitting, as previous studies that have employed this glove model have pointed out [13]. Nevertheless, the improper glove fit effect has not been analyzed in depth. In addition, to locate both PIP and DIP gauges, they are slightly inclined and partially run in parallel (see Figure 2). This gauge arrangement might cause overlapping problems for specific hand sizes if a given gauge physically covers both PIP and DIP joints. The aim of this work is to report the various problem types that might arise when using the 22 DoF CyberGlove with different hand sizes and determining the appropriate hand size to record hand kinematics with these gloves. The main purpose of this work is to offer a piece of advice to future data glove users/manufacturers. 

## 2. Materials and Methods

Experiments were performed in two different phases. The first one consisted in determining the appropriate hand size recommended for recording without problems when using these gloves. The second phase consisted in validation by recording subjects whose hand size fell within the limits obtained previously while performing ADLs. One right and one left CyberGlove III with 22 DoF were used to acquire data in both the experiment phases. Only the subjects’ dominant hand data were considered in all the recordings. All the experiments were approved by the Universitat Jaume I Ethics Committee (approval reference number CD/31/2019). The participants were previously informed about the characteristics of the experiment and provided their written consent. 

### 2.1. Phase I: Determining the Appropriate Hand Size

Six healthy adult subjects, selected as being representative of the adult population in hand size and laterality terms (3 males, 3 females, all right-handed; hand length from 172 to 196 mm, considering hand length as the distance between the distal wrist crease and middle finger’s fingertip), volunteered to participate in the experiment. Hand size variety was prioritized over sample size during subjects’ recruitment, as the aim of the experiment was only the analysis of glove functioning in different hand sizes, checking the data isolated for each specific hand length. 

**Figure 3:**
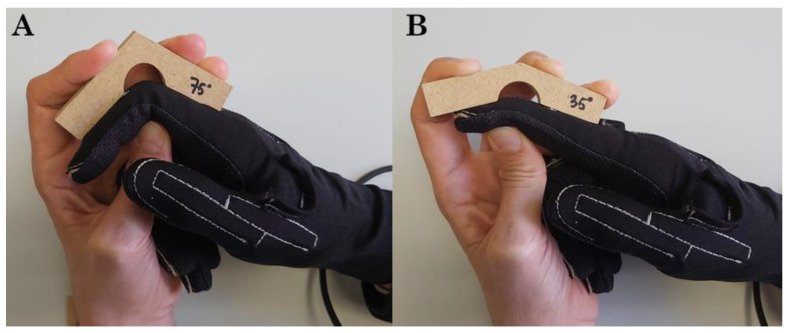
Controlled static postures recorded using wooden pieces. (**A**) Controlled static postures recorded using wooden pieces of 75°. (**B**) Controlled static postures recorded using wooden pieces of 35°.

First, any problems caused by overlapping gauges were checked by measuring the controlled static postures of the PIP and DIP joints by using wooden pieces of 35° and 75° (Figure 3). To control flexion, pressure was applied on both the palmar and dorsum sides of the finger, as Figure 3 shows. When recording a finger’s PIP joint, the same finger’s DIP was controlled to have no flexion, and vice versa. Recordings were taken for index to little finger, and the raw data values recorded from strain gauges (between 0 and 255, with the highest values indicating a more flexed angle) were analyzed. If one strain gauge also ran over its adjacent joint, the raw data value would increase, although the joint remained in the neutral position if the adjacent joint flexed. This was checked for each hand size. 

Second, recordings were also taken during motion tasks to look for any other problems that could arise from improper fitting under more realistic conditions. Subjects were asked to perform a free movement task. It consisted in closing and opening their fist, and grasping a computer mouse and a pen (both performed with their dominant hand). Scatter plots of the raw data recorded for each hand length were performed, and extreme raw data values (close to 0 or 255), implying non-achievable DIP joint angles, were identified. Their relation to bad glove fitting was discussed. 

### 2.2. Phase II: Validation

Ten healthy adult subjects (male, 7 right-handed and 3 left-handed, hand length from 184 to 207 mm) volunteered to participate in the experiment. The minimum hand length required to participate was 184 mm because the minimum recommendable hand length for using the 22 DoF CyberGlove was observed to be about 184 mm after performing the Phase I experiments. Again, hand size variety (in this case above 184 mm) was prioritized over sample size during subjects’ recruitment, as the aim of the experiment was analyzing glove functioning in different hand sizes, checking the data isolated for each specific hand length. 

**Figure 4:**
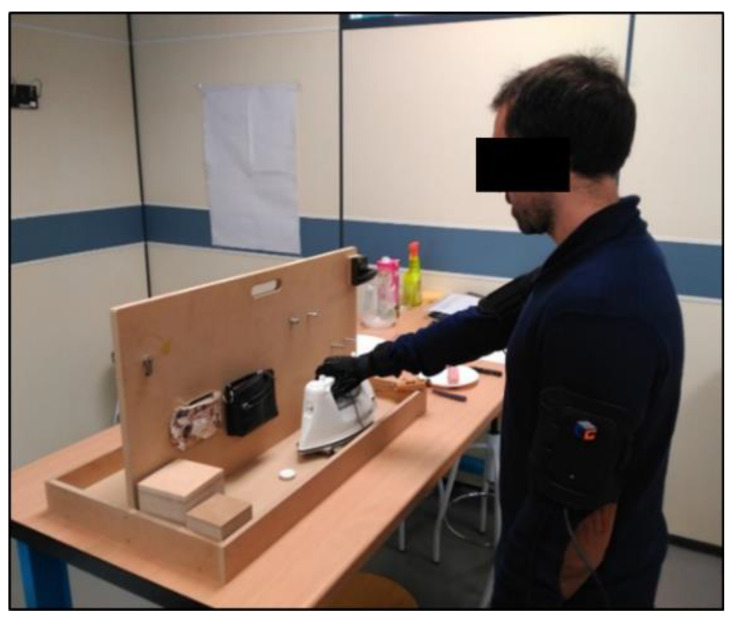
A participant performing the Sollerman Hand Function Test while wearing the 22 DoF CyberGlove.

The validation procedure consisted in checking the participants’ functional range of motion (FROM) while performing a set of representative tasks of ADLs and wearing data gloves on their dominant hand (Figure 4) against the FROM reported in the literature. The 20 Sollerman Hand Function Test (SHFT) [14] tasks were considered, together with 6 other ADLs to include the performance of the grasp types underrepresented in SHFT (intermediate, special pinch, and non-prehensile) according to the real frequency of grasps in ADLs [15]. The SHFT was originally conceived as a functionality test representative of the most commonly performed ADLs and grasp types, considering tasks such as opening a door, using a telephone, taking coins from a purse, or serving water from a jug, among others (see [14] for a complete description of the tasks). All the tasks were carried out using the dominant hand, except for some specific tasks that were performed with both hands according to the test instructions. A description of tasks can be found in the original article that presents the test [14]. Joint angles were computed according to a previously validated calibration protocol [7]. This calibration protocol uses: (i) a manual method for the calibration of all interphalangeal joints and for the thumb metacarpophalangeal joint, based on the recording of controlled static postures, (ii) a closed-loop kinematic method for the calibration of the thumb carpometacarpal joint, based on closed-loop dynamic trials, and (iii) a combination of a manual method (with controlled static trials) and an open-loop kinematic one (with controlled path dynamic trials) for the calibration of fingers’ metacarpophalangeal joints. The FROM of the DIP joints was computed for each subject as the difference between the 5th percentile and the 95th percentile of the recorded angles. Box and whisker plots of the recorded joint angles were built and related to subjects’ hand size.

## 3. Results and Discussion

### 3.1. Phase I: Determining the Appropriate Hand Size

**Figure 5:**
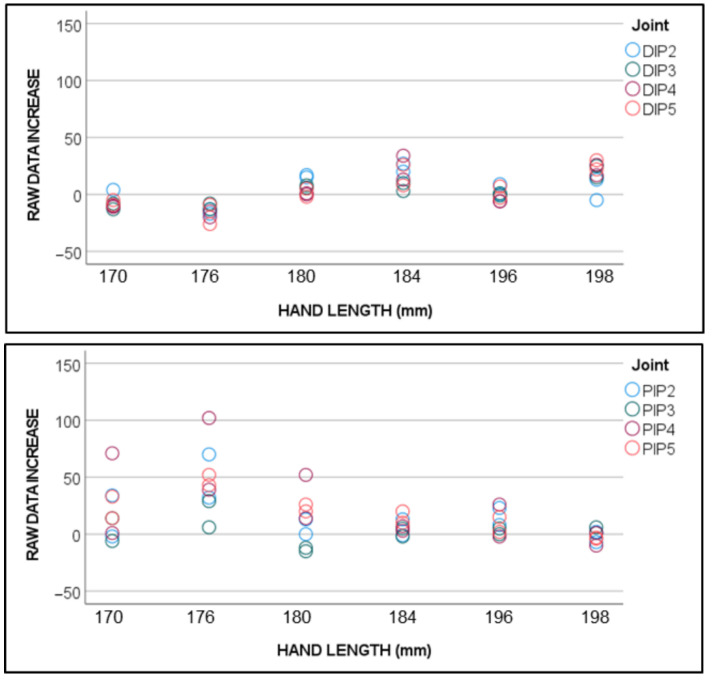
Increases in the raw data values in gauges, which were assumed to remain in flexion = 0. Color legend: index finger DIP (DIP2) in blue, middle finger DIP (DIP3) in green, ring finger DIP (DIP4) in red, and little finger DIP (DIP5) in orange.

Figure 5 presents the raw data value increases (regarding their neutral value when flexion = 0) of the gauges, which were assumed to remain with no flexion during the static recordings of phase I.

**Figure 6:**
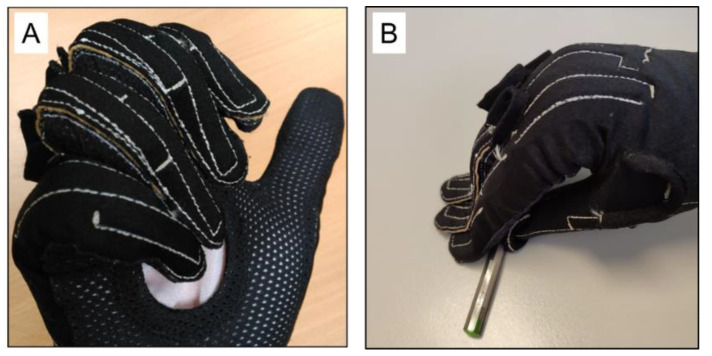
(**A**) PIP sensors spanning on PIPs and DIPs. (**B**) Fingertips hindering manipulation and bending.

In small- and medium-sized hands (length under approximately 184 mm), the flexion at the PIP joints was recorded during the DIP-controlled flexion trials, when the PIP joints were not actually flexed. Conversely, this effect was less frequent when recording the PIP static postures while maintaining DIP in the neutral position. This occurred because gauges were too large, and PIP gauges spanned both the PIP and DIP joints (they physically overlapped the glove). This means that they can record the cumulative flexion of adjacent joints (Figure 6A).

**Figure 7:**
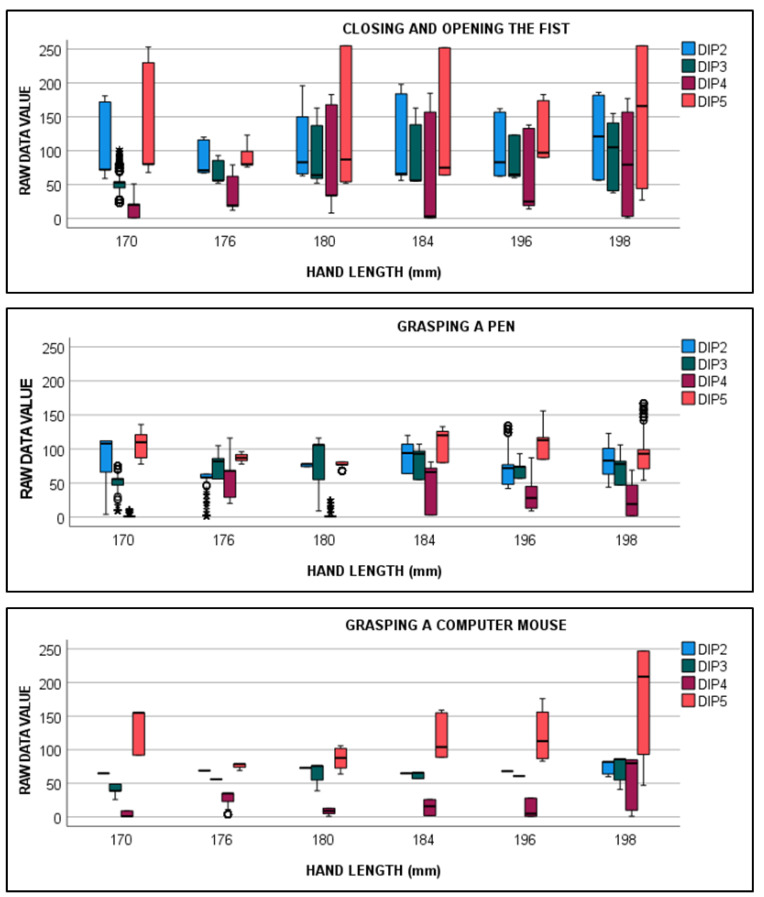
Raw data recorded during the three motion tasks for each hand length. Color legend: index finger DIP (DIP2) in blue, middle finger DIP (DIP3) in green, ring finger DIP (DIP4) in red, and little finger DIP (DIP5) in orange.

The raw data values recorded by each gauge while performing the grasping tasks and closing–opening fists are presented in Figure 7. As previously stated, these values can vary from 0 to 255, and they are higher when the joint is flexed and lower when it is extended. During these recordings, the mean neutral value (flexion = 0) across subjects was 55 for the index and middle DIP, 8 for the ring DIP, and 70 for the little finger DIP. 

Figure 7 shows that extreme (non-natural) extension values (raw data values close to 0) were recorded while opening and closing fists and for the grasping tasks, especially in hand lengths shorter than 184 mm. An inspection found that these values were attributable to the glove badly fitting hands, and it bent when it came into contact with an object or surface (Figure 6B).

Furthermore, the little finger DIP gauge presented higher raw data values than the others in the neutral position. This offset value is defined by the manufacturer to ensure that the gauge signal does not reach values of 0 or 255 when flexing the joint within its range of motion. Nevertheless, the offset value of the little finger DIP gauge was higher than that observed in the other gauges. This was why the signal occasionally saturated at 255 (especially with small hands because, apart from this, the gauge was recording both the PIP and DIP flexions).

From these results, the minimum recommendable hand length for using the 22 DoF CyberGlove was established at around 184 mm, determined as the minimum hand size of all the subjects who did not show extreme values during experiments. This size corresponds to a 23rd percentile for men and to an 81st percentile for women according to the anthropometric data of the US adult population from the PeopleSize software (OpenErgonomics, Wroxham, UK). Nevertheless, no fitting problems were observed for subjects’ hand width because the elastic fabric of the main CyberGlove body offers a tight palm fit.

### 3.2. Phase II: Validation of the Obtained Minimum Hand Length

**Figure 8:**
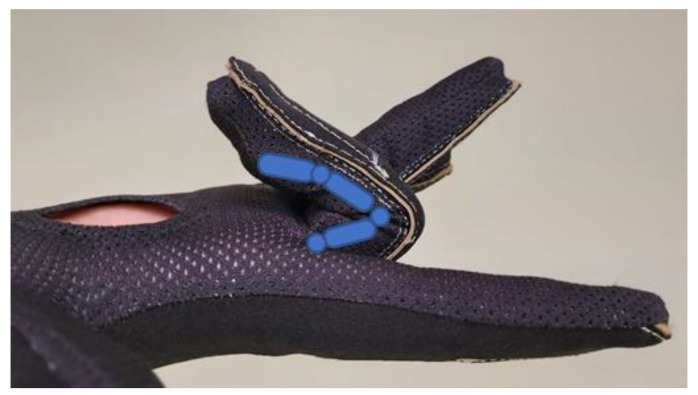
Middle finger DIP (represented in blue) flexed without bending the corresponding gauge.

Table 1 presents the median FROM of each finger for all the subjects when they performed the SHFT. The FROM for the index DIP joint was lower than the mean DIP FROM reported in the literature while performing SHFT [16] (43.35° vs. 57°), and the little DIP FROM was higher (71.08° vs. 59°). This difference in the DIP FROM index may be attributed to two main factors: (1) a very tight glove that hinders mobility, which was reported by one male subject with a hand length of 193 mm (54th percentile) and a palm width of 95 mm (90th percentile) (for the US adult male population, data from the PeopleSize software (OpenErgonomics, Wroxham, UK)), and (2) very thin fingers. This means that the fingers move inside the glove without bending gauges (Figure 8). Contrarily, the increase in the little finger DIP FROM can be attributed to an occasional simultaneous measurement of both the PIP and DIP joints. This problem may occur because the gauge size and the overlapping distance of the PIP and DIP gauges in parallel are the same in all fingers, but the distance between the PIP and DIP joints is obviously longer in index fingers than in little fingers.

**Figure 9:**
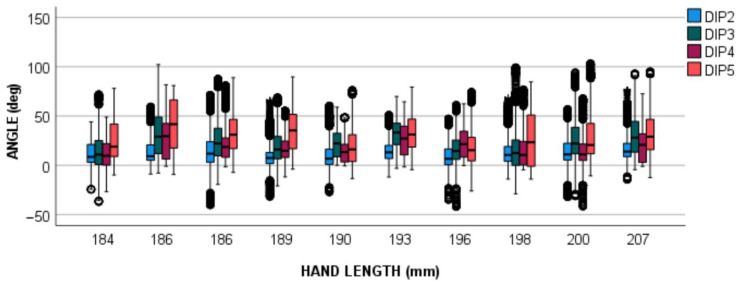
Recorded DIP angles for each subject and finger. Each subject was labeled with his/her hand size (in mm).

The boxplot of all the angles recorded while performing the SHFT is presented in Figure 9, along with subjects’ hand size. This evidenced that the recorded angles were not affected by hand size when it was longer than 184 mm and the glove properly fitted.

Apart from this, subjects reported a lack of touch sensitivity while performing the SHFT. This was obviously attributed to the fact that both hand fingertips were covered by the glove (contrarily to the 18 DoF glove) (Figure 2). 

## 4. Conclusions

As previously stated, the aim of this work was to provide a piece of advice to data glove users or future data glove users/manufacturers. Our experiments evidenced that a minimum hand length of 184 mm is required to properly record DIP joint angles with the 22 DoF CyberGlove. Nevertheless, when validating these results, although the glove properly fits, some problems may appear, such as the difficulty to record small flexions or a lack of touch sensitivity. Therefore, we conclude that more glove-sizing information is needed when acquiring a data glove with sensors to measure DIP joints. A large-sized data glove (such as the 22 DoF CyberGlove) should not be used to study the kinematics of a sample of subjects who are intended to be representative of the adult population as far as hand sizes and gender are concerned. Data glove usability would significantly improve if two or three different glove sizes were commercially available, or if the position/size of gauges was reconsidered during glove designing, and if thinner materials were used to tailor the main glove body. Moreover, the offset value of the raw data taken when gauges are in a neutral position should be checked by manufacturers before distributing gloves to prevent raw data signal saturation when gauges are flexed within joints’ ranges of motion. Finally, some of the problems identified might be lessened by using more complex non-linear reconstruction methods, such as those based on big data, e.g., neural networks.

The findings can be summarized as a low reliability of the recorded flexion in PIP and DIP joints in hand lengths under 184 mm, being inconvenient for applications requiring precision, such as hand kinematics analysis, functional assessment, or robotics/prosthetics, but neglectable in other applications such as gaming or virtual reality. Nevertheless, the lack of touch sensitivity (and therefore, tactile feedback) may suppose a problem in any manipulative application.

## Figures and Tables

**Table 1:** Recorded DIP joints for FROM during the Sollerman Hand Function Test (SHFT) of subjects’ dominant hand vs. the reported values in the literature.

Joint	Mean Recorded from SHFT ± SD (P5/P95)	Mean Reported from SHFT in [16]
Index DIP	43.35° ± 6.99° (−3.9°/39.44°)	57°
Middle DIP	60.95° ± 10.21° (−0.04°/60.91°)	54°
Ring DIP	50.38° ± 7.23° (−1.02°/49.36°)	45°
Little DIP	71.08° ± 14.71° (−0.73°/71.81°)	59°

## Data Availability

Experimental data publicly available at Zenodo (https://doi.org/10.5281/zenodo.6548190).
